# Direct Evidence That Microplastics Are Transported to the Deep Sea by Turbidity Currents

**DOI:** 10.1021/acs.est.4c12007

**Published:** 2025-04-04

**Authors:** Peng Chen, Ian A. Kane, Michael A. Clare, Euan L. Soutter, Furu Mienis, Roy A. Wogelius, Edward Keavney

**Affiliations:** † Department of Earth and Environmental Sciences, University of Manchester, Manchester M13 9PL, United Kingdom; ‡ School of Earth Sciences and Resources, China University of Geosciences, Beijing 100083, China; § Ocean BioGeoscience, National Oceanography Centre, Southampton SO14 3ZH, United Kingdom; ∥ Department of Ocean Systems, Royal Netherlands Institute for Sea Research (NIOZ), Den Burg 1790 AB, Netherlands; ⊥ School of Earth and Environment, University of Leeds, Leeds LS2 9JT, United Kingdom

**Keywords:** microplastic transport, turbidity current, ocean sediment, deep-sea monitoring, submarine canyon

## Abstract

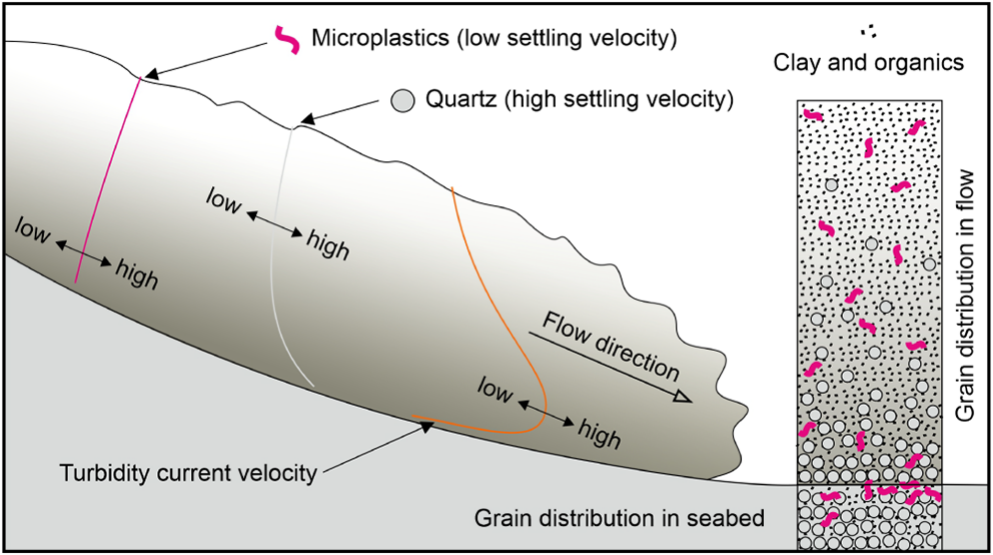

Microplastics pervade the global seafloor, yet the mechanisms by which this pollutant is increasingly transported to the deep sea remain unclear. Fast-moving sediment avalanches (called turbidity currents) are hypothesized to efficiently transport microplastics into the deep sea. However, while this has been inferred from field sampling of the seafloor, it has never been demonstrated outside of a laboratory setting. Here, we provide direct field-scale evidence that turbidity currents in submarine canyons not only transport globally significant volumes of mineral and organic matter into the deep sea but also carry large quantities of anthropogenic particles, including microfibers and microplastic fragments. In situ hydrodynamic monitoring, coupled with direct sampling of the seafloor and material suspended by turbidity currents, reveals that even a submarine canyon whose head lies hundreds of kilometers from land acts as an efficient conduit to flush sediment and pollutants from the continental shelf to water depths greater than 3200 m. Frequent and fast turbidity currents supply oxygen and nutrients that sustain deep-sea biodiversity and fishing grounds in, and adjacent to, such canyons. Our study therefore confirms that these biodiversity hotspots are colocated with microplastic hotspots, indicating that the more than 5000 land-detached canyons worldwide can be important but previously unproven conveyors of anthropogenic pollution to the deep sea.

## Abstract 2

This study presents direct field-scale evidence that turbidity currents are significant agents for transporting microplastics into the deep sea.

## Introduction

All environments on Earth are polluted by microplastics.^1^ Oceans are the ultimate repository for most of this pollution.^2−4^ The effects of plastic pollution on marine ecosystems and the implications for human health are of growing concern, as more than ten million tonnes of plastic enter the global ocean each year, with the seafloor being a globally important sink for plastics.^4−6^ Microplastics represent an important proportion (13.5%) of the global marine plastic budget^7^ and occur as small (<1 mm) fibers from synthetic textiles,^8^ fragments^9,10^ and manufactured particles,^11,12^ or fragments derived from the breakdown of larger plastic debris.^13^ In addition, anthropogenically modified natural microfibers may be equally persistent in the environment as plastic microfibers.^14^ Due to their small size, microfibers and microplastics can be ingested by organisms across all trophic levels, enabling the transfer of harmful toxic substances coating or leaching from them.^9,15,16^ Characterizing the physical controls on microplastics transport and the effectiveness of their burial once deposited on the seafloor is therefore critical to understanding their distribution, their bioavailability, and, hence, the potential threats to globally important seafloor ecosystems in the deep sea.^17−21^

Oceanic gyres are responsible for concentrating the estimated 1% of the ocean plastic budget that is found on the ocean surface, in so-called “ocean garbage patches”.^2,3^ The remaining 99% resides in the deep sea, on and within sediments below the seafloor.^15,22^ Microfibers and microplastics that have been sampled on the deep seafloor are preferentially concentrated within distinct physiographic settings, rather than corresponding to the extent of overlying surface garbage patches, indicating that their distribution cannot be accounted for by vertical settling alone.^19−21^ It has been shown that relatively weak seafloor currents driven by global thermohaline circulation can concentrate microplastics into seafloor hotspots in a similar way to their surface counterparts;^21^ however, the primary pathways of microplastics to the deep sea have only been hypothesized based on their preferential occurrence within distinct physiographic settings (i.e., canyons and deep-sea trenches) or inferred based on laboratory-scale experiments.^23,24^

Density-driven, sediment-laden seafloor flows known as turbidity currents that “flush” submarine canyons^25^ have been hypothesized to also carry microplastics.^19,26,27^ Turbidity currents transport and sequester vast amounts of land-derived natural sediments,^28^ organic carbon,^29^ and pollutants.^30^ These voluminous, powerful, and often destructive flows typically originate on or near the continental shelf edge and transfer sediment through submarine canyons directly or indirectly connected to rivers, or through land-detached canyons fed by river-derived and coastal sediment transported vast distances along the continental shelf by ocean currents.^31^ Thus, microplastics supplied by polluted rivers may plausibly be carried from continental shelves to the deep sea via turbidity currents passing through submarine canyons.^32−34^ However, a paucity of direct deep-sea monitoring and in situ sampling means that the role of turbidity currents in microplastic transport has never been definitively demonstrated.

**Figure 1:**
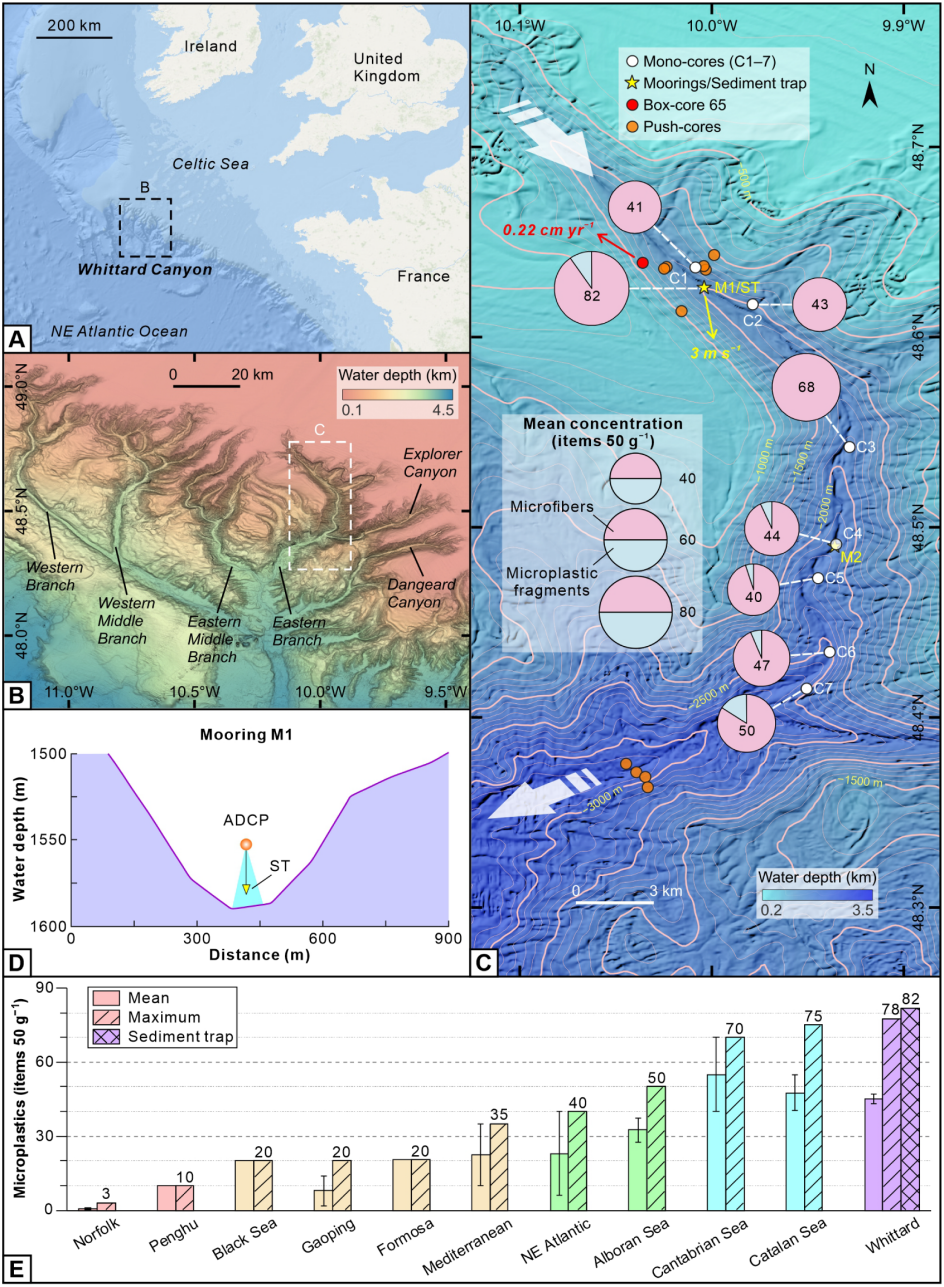
Large volumes of microplastics distributed on the seafloor of the land-detached Whittard Canyon. (A) Location of the Whittard Canyon, which is separated from the closest coastline by 300 km of continental shelf. (B) Overview of the four branches of the Whittard Canyon and the adjoining Explorer and Dangeard Canyons. (C) Two moorings (M1 and M2), the sediment trap (ST), the seven mono-cores (C1–7), the box-core 65, and the push-cores in the eastern branch of the Whittard Canyon. Mean concentrations and relative percentage of microfibers and microplastic fragments at each mono-core are also shown. (D) Schematic figure showing the ADCP and ST at M1 indicated in (C). (E) Comparison of microplastic abundance in different submarine canyons worldwide (see Table S1); different color groups highlighting the variation of maximum microplastic concentrations; and error bars showing standard error of the mean. The sediment trap is a single sample and yielded the highest microplastic concentration.

Here, we address this important knowledge gap and provide the first field evidence showing that turbidity currents transport microplastics from shallow continental shelves to the deep sea and that deposits sequester part of their anthropogenic load within submarine canyons. We achieve this by integrating in situ monitoring and direct sampling of turbidity currents (Figure S1) with high-resolution seabed mapping (Figure S2) and analyzing microplastics from seafloor samples taken from 1417 to 3270 m water depth (Figure 1). The Whittard Canyon lies in the Celtic Sea, in the Northeast Atlantic Ocean. During the last ice age, the canyon was river-connected,^35^ but during sea-level rise, it evolved into a land-detached canyon with its head approximately 300 km from the present-day shoreline (Figure 1A). The Whittard Canyon system has four main tributary branches (Figure 1B), which connect with the broad shelf at approximately 200 m water depth and merge at 3500 m into the wider Whittard Channel, leading to the Celtic Fan at 4500 m water depth.^36^ The Whittard Canyon is an ideal study area because (i) its physical dimensions and grain-size are broadly comparable to many canyons worldwide;^31,36^ (ii) ocean circulation patterns and velocities are well-constrained, making global comparisons possible;^36^ (iii) the Whittard Canyon is prone to frequent turbidity currents, despite being disconnected from any direct river input;^31^ (iv) high levels of microplastics have been reported from the adjacent continental shelf;^37^ and (v) high-resolution seafloor and near-seafloor monitoring data provide the necessary spatial and temporal context to investigate our key questions. Using these data, we addressed three questions. First, do turbidity currents carry microplastics? Second, how does the spatial distribution of seafloor microplastics vary along a land-detached canyon affected by turbidity currents? Finally, given their generally low settling velocity, how efficiently and where are microplastics sequestered into seabed sediments?

## Materials and Methods

### In Situ Monitoring and Direct Sampling of Turbidity Currents

**Figure 2:**
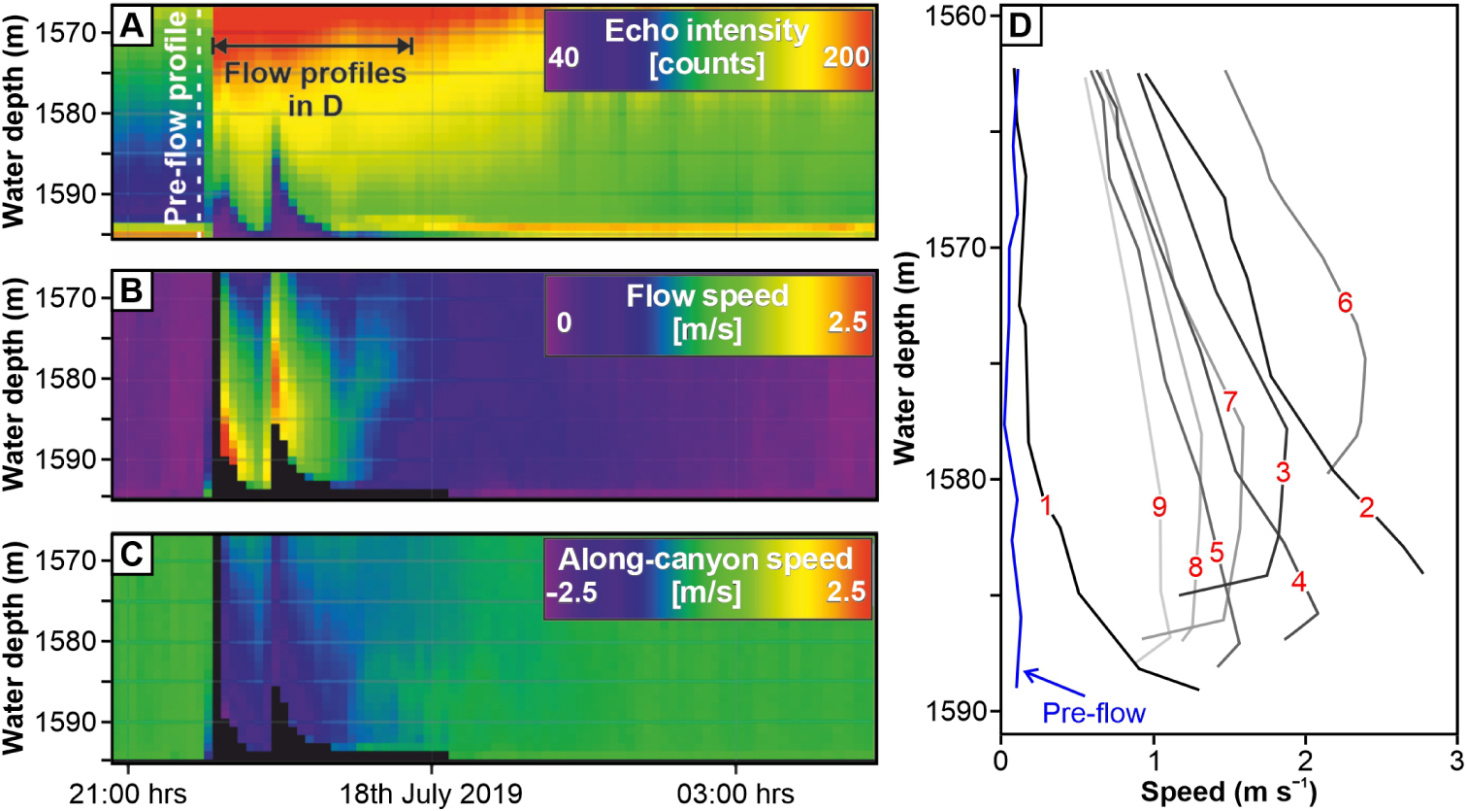
Monitoring data showing the passage of the turbidity current determined to have filled the sediment trap at mooring M1, at 21:45 on 17 July, 2019. (A) Echo intensity giving an indication of sediment concentration within the turbidity current. (B) Turbidity current flow velocity, revealing two distinct peaks; black areas indicate signal attenuation by sediment load. (C) Along-canyon speed between moorings M1 and M2 (negative values indicate down-canyon velocities). (D) Velocity profiles recorded every ten min with the darkest profile (no. 1) indicating the onset of the flow and the lightest profile (no. 9) indicating the last in the series.

Near-seafloor hydrodynamic monitoring was performed from June 2019 to August 2020 using a 600 kHz downward-looking Acoustic Doppler Current Profiler (ADCP) mounted 30 m above the seafloor on a deep-water mooring (M1; 1591 m water depth, 26 km downstream of the canyon head at 48.626° N, 10.004° W) in the eastern branch of the Whittard Canyon (Figure 1). The ADCP recorded vertical profiles of water column velocity and acoustic backscatter (a proxy for sediment concentration), capturing data at 1 m intervals every 5 min (Figure 2). An additional ADCP mooring (M2; 2259 m water depth, 21 km downstream from M1, 47 km downstream of the canyon head at 48.490° N, 9.936° W) was deployed 14 m above the seafloor to record currents at lower resolution, measuring every hour across 16 m vertical intervals. M1 was also equipped with a McLane Parflux sediment trap (ST) mounted 10 m above the seafloor (Figures 1 and S1). This ST consists of an upward-facing funnel (made of high-density polyethylene) overlying a mechanical carousel that rotates every 18 days to present a new 500 mL sampling bottle. The sediment collected from the first sampling bottle, representing the sedimentation of the first turbidity current within the initial 18 days,^31^ weighed 553.2 g; a 58.5 g subsample was taken for microplastic extraction and grain-size analysis.

### Seafloor Sediment Samples

A mono-corer was used to sample seafloor and shallow subseafloor sediments (Figure S1). After retrieval, cores were immediately sliced into cm slices. Half of each slice was stored for sedimentological analysis at −20 °C, while the other half was preserved for plastic analysis and stored at 4 °C in aluminum foil. Seven mono-core samples, numbered C1 to C7, were collected from below the head of the canyon to just north of the eastern branch of the Whittard Canyon and analyzed. These samples were taken along the canyon axis from 1421 to 2683 m water depth (Figure 1C) and subsampled at 1 cm vertical intervals, yielding up to 10 subsamples per core, depending on core recovery. Only C3 contained 3 subsamples, while the other six mono-cores each had 10 subsamples, resulting in a total of 63 subsamples collected (Table S2). In addition, ten push-core samples, which similarly recovered seafloor and subseafloor sediments, were collected from 1417 to 3270 m water depth and analyzed (Figure 1).

### Microplastic Extraction, Identification, and Quantification

**Figure 3:**
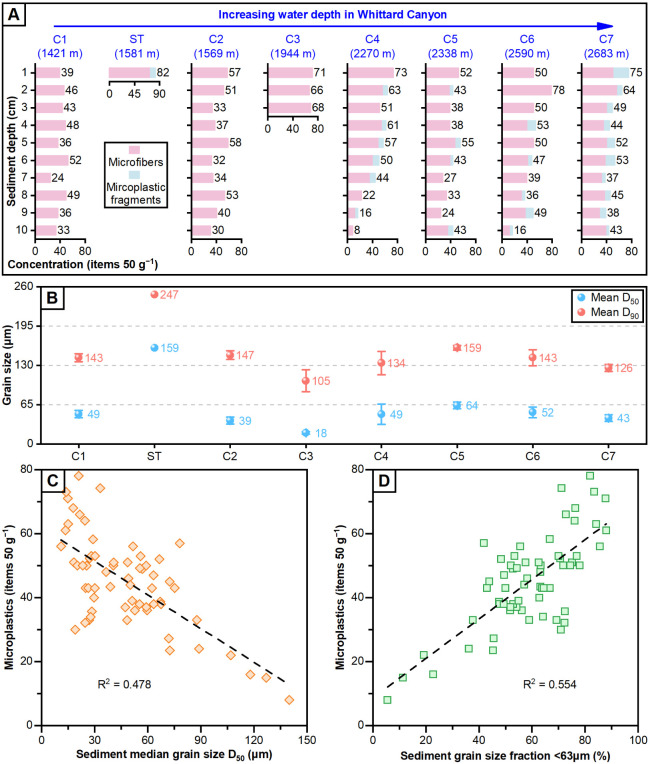
Variations in microplastic concentration and the grain size of host sediment. **(A)** The total concentrations with relative percentage of microfibers and microplastic fragments in the seven mono-cores (C1–7) and the sediment trap (ST). (B) The mean D_50_ and D_90_ variations of C1–7 and ST, with error bars showing the standard error of the mean. **(C and D)** Correlation of microplastic concentrations with median grain size (D_50_) and fine sediment fraction (<63 μm) of each sediment sample.

The 1 cm sediment core horizons had variable weights and water content, so samples were dried overnight in a drying oven set to 50 °C. The dried samples were weighed, and for comparative purposes, the weight and microplastic content quoted were normalized to 50 g. Sediment samples were then stored in glass beakers and covered with aluminum foil. Samples were added to a 1 L glass beaker with approximately 700 mL of a dense ZnCl_2_ solution (1.7 g cm^–3^) and disaggregated using a magnetic stirrer and mixed until the sediment/ZnCl_2_ solution was homogenized. The microplastics were extracted from the sediment using a polyvinyl chloride Sediment Microplastic Isolation (SMI) unit following a protocol developed for microplastic extraction^38^ and modified to avoid polyvinyl chloride contamination.^39^ The solution was added to the SMI unit, and the beaker was rinsed with the ZnCl_2_ solution to flush any remaining sediment/microplastic. Prior to each use, the SMI unit was disassembled and thoroughly rinsed with Class 1 Milli-Q deionized water. Following settling overnight, the headspace supernatant was isolated by closing the ball valve of the SMI unit and rinsing with extra ZnCl_2_ solution to flush any remaining microplastics before vacuum filtering over a Whatman 541, 22 μm filter paper. The filter paper was then placed in a labeled Petri dish and covered. Throughout the duration of the microplastic extraction procedure, all individuals wore white, cotton laboratory coats and latex gloves. All of the microplastic extraction stages were performed in a clean laboratory in a fume cupboard. When the sediment samples were mixed in the 1 L glass beaker and settled in the SMI units, they were covered with aluminum foil to limit airborne microplastic contamination. When it was not possible during the sample preparation to cover the sediment sample with aluminum foil, an open Petri dish with a blank Whatman 541, 22 μm filter paper was placed in the fume cupboard and used as a contamination control procedural blank (Table S3). The prepared filter papers, both from the sediment extraction process and the airborne contamination control blanks, were analyzed in a clean microscopy laboratory using a Zeiss Axio Zoom V16 stereomicroscope at 20–50× magnification. Filter papers were traversed systematically to identify microplastics based on the following criteria: (1) no visible cellular or organic structures, (2) a positive reaction to the hot needle test,^40,41^ and (3) maintenance of structural integrity when touched or moved. Visual sorting is highly dependent on the observer’s performance and is challenging for particles <500 μm, with significant errors below 300 μm. Therefore, we retained results only for particles >300 μm. Microplastics were categorized based on their color and shape, i.e., whether they were microfibers, microplastic fragments (including films), or microbeads (Figure S3). The percentage of different microplastic shapes in each sample (Figure 3A) and the mean microplastic concentration of each sample location (i.e., ST, C1–7) were also calculated and presented (Table S4).

### Polymer Identification

Industry produces a wide variety of plastic polymers that break down when exposed to weathering processes. A key reaction in this breakdown is the oxidation of reduced carbon, which increases the quantity of oxygen-bearing functional groups in the plastics. This increase can serve as an indicator of degradation: the more oxygen present, the more degraded the plastic.^42^ This degradation process is similar to how natural organic carbon materials in sedimentary rocks break down, where oxidative processes convert pristine reduced carbon compounds like collagen or keratin into more oxidized, less polymerized fragments.^18^ Infrared methods are particularly useful for identifying diagnostic functional groups, determining the most likely original plastic type, and providing information about the degree of degradation based on compositional differences between the probable original material and the recovered sample from the field.^43^ Based on the shapes and colors identified using the stereomicroscope, microfibers and microplastics were classified into different groups, with one group selecting only one representative sample for FTIR analysis. A subset (*n* = 42) of the extracted microfibers and microplastic fragments was analyzed using a PerkinElmer Spotlight 400 micro-Fourier transform infrared (μ-FTIR) spectrometer to confirm a polymer (or other) origin. The FTIR spectrum range was set at 4000–650 cm^–1^, with a resolution of 4 cm^–1^ at a rate of 16 scans per analysis. Data were processed, and diagnostic functional groups were identified using the PerkinElmer Spectrum IR and Spectrum IMAGE software with a standard reference library to assign polymer type and assess the degree of degradation. Microplastic polymers were consequently confirmed based on the library comparison results with >70% confidence (Figure S4).

### Sediment Grain-Size Analysis

All sediment samples were analyzed using a Malvern Mastersizer 3000 equipped with an automated wet dispersion unit (Hydro LV). The samples were subjected to a small amount of ultrasonic treatment and premeasurement dispersion. Three aliquots were analyzed to ensure that each sample was completely dispersed. Each measurement was replicated five times, with a coefficient of variance (COV) below 3% for D_50_ and below 5% for D_10_ and D_90_, and the average of the five valid results was finally reported. The grain-size distribution (Figure S5), indicating the volume percentage of grains in a certain size interval,^44^ was constructed. Some grain-size percentiles, such as D_10_, D_50_, and D_90_, were exported from the software. Further statistical analysis between microplastic concentration and grain-size percentiles was conducted (Figure 3B–D).

### Sedimentation Rate of Seafloor Sediments

The ^210^Pb dating technique is based on alpha spectrometry from ^210^Po and is used to infer particle accumulation rates over the past 100 years.^45^ The ^210^Pb dating results are analyzed by a two-layer, 1D vertical eddy diffusion model, assuming a constant input of ^210^Pb and steady sedimentation rates.^46,47^^210^Pb samples are chosen as such to avoid sandy intervals as they generally hold lower ^210^Pb signatures than finer-grained intervals.^48^ Therefore, the finer sediments (silt with normally graded successions) of box-core 65 in the Whittard Canyon are suitable and are selected for ^210^Pb dating. Box-core 65, located at a water depth of 1105.5 m at 48.6391° N, 10.036° W, is near the mono-core C1 (Figure 1C). The total length of box-core 65 is 41.3 cm, with 12 intervals (0–0.5 cm, 0.5–1 cm, 1–1.5 cm, 2–2.5 cm, 3–4 cm, 5–6 cm, 9–10 cm, 13–14 cm, 17–18 cm, 24–25 cm, 31–32 cm, 38–39 cm) sampled. ^210^Pb activity in the sediments of box-core 65 varies from 400 to 530 mBq g^–1^ at the surface to a background activity of ∼25 mBq g^–1^ with depth. The shape of the ^210^Pb profile varies as a function of accumulation rate, diffusive mixing constant, and bioturbated mixing depth^49^ (Figure S6).

### Global Comparison of Microplastic Concentrations in Submarine Canyons

Geomorphological mapping of submarine canyons was conducted in a previous global study;^50^ a total of 5849 submarine canyons were mapped worldwide and subdivided into three main types: Type 1, shelf-incising submarine canyons having heads with a clear bathymetric connection to a major river system; Type 2, shelf-incising submarine canyons with no clear bathymetric connection to a major river system; and Type 3, blind submarine canyons incised onto the continental slope (note that Antarctic canyons are excluded due to a lack of sufficient study). Here, we define land-detached submarine canyons as submarine canyons that are not connected to a major river system, so Type 2 and Type 3 can both be included, with a total number of 5696 (Figure S7). There is a 45–55% increase in potentially active submarine canyons from just land-attached submarine canyons to land-detached submarine canyons, so the number of land-detached submarine canyons will be higher than this value. To compare measured microplastic concentrations from the samples in the Whittard Canyon with other submarine canyons worldwide, we compiled data from publications that provided details on the mean and maximum microplastic concentrations of ten submarine canyons^26,51−53^ (Figure 1E and Table S1).

### Relating Microplastics to Seafloor Shear Stress

**Figure 4:**
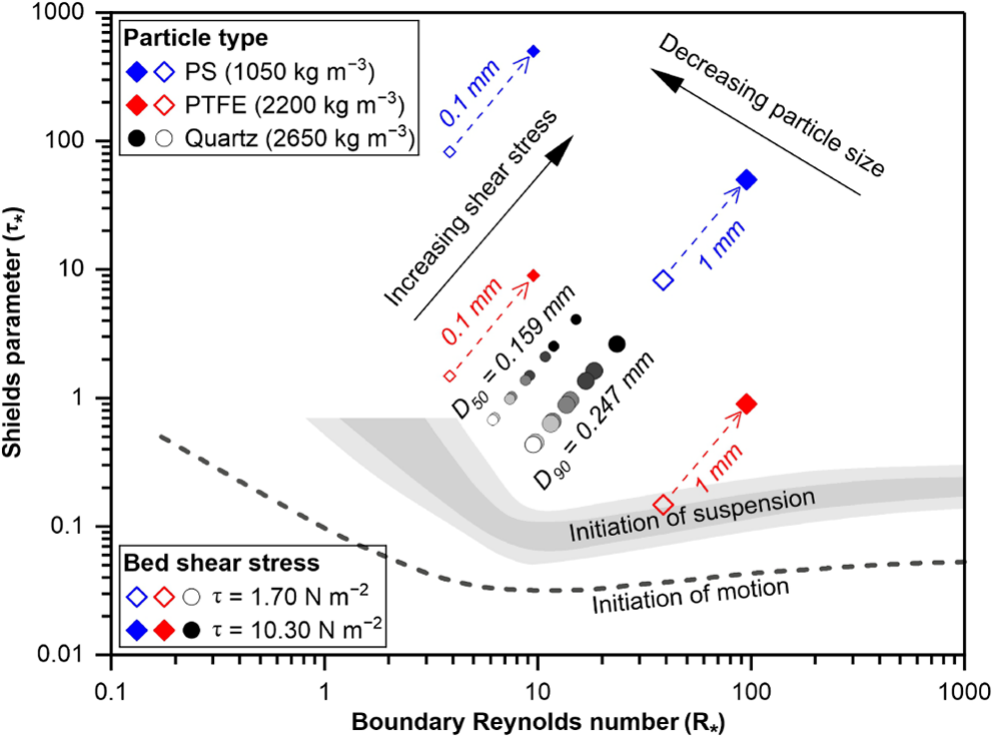
Shield’s diagram showing the condition for moving (above the dashed gray line) and suspending (within or above the gray filled area) particles. D_50_ and D_90_ of quartz grains in the sediment trap are shown, with white- to black-filled circles indicating the transition from the minimum to the maximum calculated shear stresses generated by the nine recorded turbidity current profiles (Figure 2D). PS (polystyrene, 1050 kg m^–3^) and PTFE (polytetrafluoroethylene, 2200 kg m^–3^) are the minimum and maximum microplastic densities identified. Hollow and solid symbols indicate the minimum and the maximum shear stresses generated by the nine recorded turbidity currents profiles. 0.1 mm and 1 mm represent the general microplastic size range observed. All sediments (including quartz and microplastics) are prone to be transported in suspension by the turbidity currents in the Whittard Canyon with a shear stress >1.70 N m^–2^ (Table S5).

Bed shear stress (τ) determines which sediment the flow can transport and whether the flow will pick up additional sediment from the bed or sediment will settle out of the flow.^54^ The bed shear stress (τ) is assumed to relate only to bed roughness and will remain constant for a fixed location during the flow,^54^ and it can be calculated by
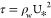
1where ρ_w_ is the seawater density (1029 kg m^–3^) and U_*_ is the bed shear velocity.^21^ In this study, the bed shear velocity (U_*_) generated by the turbidity current at the seafloor can be determined by its velocity profile, which is logarithmic between the bed and the maximum velocity:^55,56^
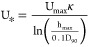
2where U_max_ is the maximum velocity, h_max_ is the height of the maximum velocity, κ is the von Kármán constant with a value of 0.4,^57^ and D_90_ is derived from the grain-size distribution in the turbidity current. The bed shear velocity (U_*_) and the resultant bed shear stress (τ) are then used to calculate the Shields parameter (τ_*_, also called dimensionless shear stress)^56−60^ and boundary Reynolds number (R_*_) for different particles using^21,55,56^
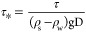
3
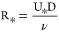
4where ρ_s_ is the particle density [quartz with 2650 kg m^–3^, polytetrafluoroethylene (PTFE) with 2200 kg m^–3^, polystyrene (PS) with 1050 kg m^–3^], g is acceleration due to gravity (9.81 m s^–2^),^56^ ν is the kinematic viscosity of seawater at 20 °C (1.0508 × 10^–6^m^2^ s^–1^) (https://ittc.info/), and D is the particle diameter (Figure 4 and Table S5).

## Results and Discussion

### Turbidity Current Activity in the Whittard Canyon

Six turbidity currents (flows 1–6) were recorded at M1 during the study period (June 2019 to August 2020), with maximum ADCP-measured velocities of 1.1–5.0m s^–1^ and estimated local velocities up to 8 m s^–1^.^31^ Here, we focus on the first of these, flow 1 (17 July 2019), as this event filled the sediment trap suspended 10 m above the seabed at 1591 m water depth, colocated at M1 (Figure 1C,D). Flow 1 occurred when the surface tidal flow was down-canyon.^31^ Flow 1 had two pulses, with maximum recorded velocities of 3 m s^–1^ and 2.5 m s^–1^, respectively, occurring toward the flow base. The acoustic backscatter signal was partially attenuated by high sediment concentrations at the onset of each of the flow pulses (Figure 2). The flow attained a thickness of at least 30 m, lasting around 3 h at M1, and was recorded at M2 (Figure 1C). The sediment comprised quartz-rich sand with a unimodal grain-size distribution, a median diameter (D_50_) of 159 μm, and D_90_ of 247 μm, with the largest grains being carbonate fragments up to 445 μm (Figure S5). The turbidity current was thus capable of carrying fine- to medium-grained sand at least 10 m above the seafloor. The plastic fishing line was observed wrapped around the M1 mooring anchor chain,^31^ demonstrating active transport of larger plastic litter through the canyon, as also shown by previous studies.^61^

### Direct Field-Scale Observation of Microfibers and Microplastics Carried by Turbidity Currents

Seafloor and sediment trap samples all contained microfibers and microplastic fragments. Additional push-cores from an across-canyon transect, 8.21 km further down-canyon from C7, also contained microplastics at water depths >3200 m (Figure 1C). 77% of the microfibers are plastic, as verified by optical microscopy and Fourier transform infrared (FTIR) spectroscopy. The most common verified polymer types include polyvinyl chloride (PVC), polyvinyl butyral (PVB), and polyethylene terephthalate (PET). The remaining 23% of microfibers are composed of semisynthetic polymers, including rayon and chlorinated rubber (Figure S4). The sediment trap sample yielded 8 microplastic fragments and 74 microplastic fibers (82 items in total) 50 g^–1^ of dried sediment; these values are comparable to the highest values recorded from seafloor sediments in submarine canyons worldwide (Figure 1E and Table S1). Sediment in the trap was collected during flow 1, revealing that this turbidity current was carrying microplastics down the canyon at a speed of up to 3 m s^–1^ as part of its sediment load. Presumably, these microplastics were supplied by the same cross-shelf transport that supplied the mineral sediment, with additional local input of discarded or lost fishing gear. Seafloor microplastic concentrations are similarly high, with up to 78 items 50 g^–1^ of dried sediment (Figure 3A). These microplastics were recorded largely within the thalweg of the canyon or slightly above it (Figure S2). This concentration is higher than that recorded in other submarine canyons globally, including land-attached canyons (Figure 1E and Table S1). It is perhaps most remarkable that such high concentrations occur in a submarine canyon that lies far from land. As there are more than 5000 land-detached canyons globally, occurring on all of the world’s continental slopes^50^ (Figure S7), the high microplastic contents reported here suggest that such canyons are globally important pathways and repositories for microplastics and that land-attached canyons, which are more efficiently connected to terrestrial outflows of pollution, may be equally, if not more, important, as demonstrated for macro-litter.^32,34,62^

### Microfibers and Microplastics Are Flushed through the Canyon to the Abyssal Plain

**Figure 5:**
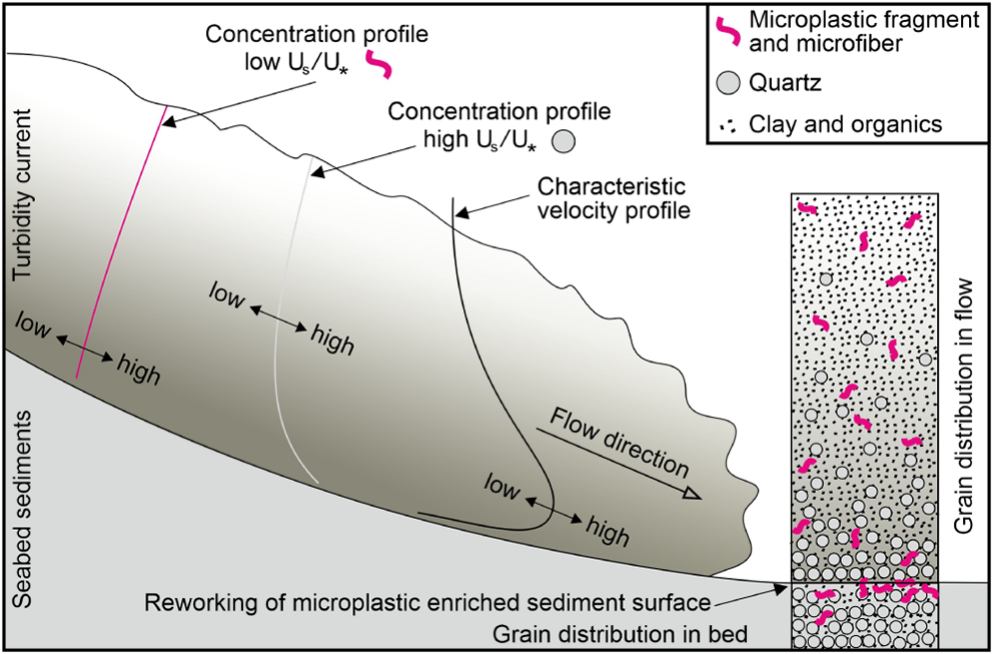
Summary figure of microplastic transport in a turbidity current. Particles with a relatively low settling velocity (U_s_), in comparison to the shear velocity (U_*_), e.g., microplastics, will tend to be more homogeneously distributed throughout the flow than those with a high settling velocity and be transported to greater water depths.

The near-seafloor flows observed during the study period exerted shear stresses capable of suspending mineral grains, microfibers, and microplastics (Figure 4). The grain size of sampled seafloor sediments was significantly finer (mean D_50_ of 18–64 μm) than that of the sediment trap (mean D_50_ of 159 μm). This is intuitive, as the canyon floor serves as the repository for finer-grained sediment within the turbidity currents and, hence, any background sedimentation. However, the mean D_90_ of the seafloor sediment samples (105–159 μm) is also considerably finer than that of the sediment trap (247 μm). This disparity suggests that, while the turbidity current carried fine-to-medium sand, this material was transported further downslope, bypassing the study area to ultimately accumulate in abyssal depths. This assertion is supported by the lack of any downslope trend in grain size (Figure 3B). A downslope decrease in grain size would be expected if flows were waning and dying out within the canyon, but this is clearly not the case. Given the significantly lower settling velocity of microfibers and microplastics compared to quartz grains,^19,63,64^ it is likely that sand suspended by turbidity currents would settle to the seafloor before microplastics (Figure 5), suggesting that microfibers and microplastics are “flushed” (sensu^25^) through the canyon toward the deep-sea submarine fan (>4500 m water depth). Other oceanic processes (e.g., offshore convection and dense shelf water cascading) could be equally or more important to the variety of sources and transport pathways for microfibers and microplastics reaching the open sea, depending on the particular setting. Selective deposition of these microplastics may occur where longer fibers become trapped during deposition^23^ or when they combine with cohesive sediment (e.g., clay flocs) to form agglomerates, decreasing their buoyancy.^65,66^

### Reworking of the Bed Enriches Seafloor Microplastic Concentrations

Samples contained mean values of 43 microfibers and 2 microplastic fragments 50 g^–1^ of dried sediment (Table S4). Most of the highest concentrations of fibers were found at 0–2 cm depth below the seafloor, with the highest being 78 fibers 50 g^–1^ of dried sediment (sample C6, 1–2 cm). In all mono-cores, there was an average 50% decrease in fiber concentrations with sediment depth from the top to the base of the core, with a maximum decrease of 87%. There was no discernible downstream trend in the number of fibers. Fragments were only found in the four furthest downstream mono-cores, with a maximum of 23 fragments 50 g^–1^ of dried sediment found in the deepest mono-core (sample C7) in the 0–1 cm layer (Figure 3A). Given the active high-energy sedimentary environments of the canyon floor, it is extremely unlikely that sedimentary depth corresponds to a monotonic increase in age; however, ^210^Pb dating of box-core 65 indicates an average sedimentation rate of 0.22 cm yr^−149^ (Figures 1C and S6), so the 10 cm sample depth is likely to be entirely within the period of plastic production (i.e., since the 1950s). Deep tidally driven currents, which have been shown to attain velocities of ± 0.6 m s^–1^ within the Whittard Canyon, i.e., up- and down-canyon,^67^ also likely affect the (re)distribution of microfibers and microplastics. Microfiber and microplastic concentrations correlate inversely with sediment D_50_ but positively with the increasing proportion of sediment grain size below 63 μm (Figure 3C,D). Finer sediments may more easily trap and concentrate microplastics in the surficial layers, owing to cohesion and lower porosity,^68^ as microplastics have been shown to infiltrate deeper into coarse sandy sediments than fine silty sediments.^69,70^ This may result in the seabed or upper layers being relatively enriched with microfibers and microplastics (Figure 5). Compared with other studies on marine sediments,^71^ the relatively weak correlations of microplastics to grain size identified here may highlight the complex near-seafloor hydrodynamics that operate within submarine canyons, including internal tides, turbidity currents, erosive events, and vertical settling, in addition to other natural processes (e.g., bioturbation^49^) and human activities (e.g., fishing adjacent to the canyon), which could overprint the relationship between microplastic concentration and sediment grain size.

### Environmental Implications

Large and powerful turbidity currents transport microfibers and microplastics into the deep sea from shallower continental shelves and are important controls on the transfer of microplastics to deep-sea sediments. We provide the first direct field evidence that active turbidity currents (Figure 2), even in a land-detached submarine canyon, transport high volumes of microfibers and microplastics (Figure 1), and that this has led to the high concentrations recorded on the seabed, with their distribution controlled by turbidity current volume, velocity, and concentration. The throughgoing nature of the turbidity currents in the Whittard Canyon, demonstrated by direct hydrodynamic monitoring and grain size trends (Figure 3), shows that microplastics are transported through the entire canyon reach, with much higher concentrations envisaged on the deeper abyssal plain at >4500 m water depth. Sediment cores reveal that microplastic concentration decreases with depth in the sediment, suggesting that microplastics may be prone to reworking on the seafloor (Figures 4 and 5) and subject to deep tidally driven currents and downslope transport.^31,67^ While the environmental risks of microplastic pollutants in aquatic systems have been well-documented,^72^ this new understanding will aid the monitoring of mitigation strategies and highlight the risk posed to deep-sea biodiversity hotspots that are also fed by nutrients and oxygen supplied by the same currents that convey microplastic pollutants to the deep sea.

## Data Availability

The bathymetry of the North-East Atlantic Ocean is derived from the Esri Ocean Basemap (https://www.arcgis.com/apps/mapviewer/index.html?webmap = 67ab7f7c535c4687b6518e6d2343e8a2). The Digital Terrain Model data for the Whittard Canyon is based on the 2022 EMODnet digital terrain model (DTM) (https://doi.org/10.12770/ff3aff8a-cff1-44a3-a2c8-1910bf109f85), which has a resolution of 1/16 × 1/16 arc minute of longitude and latitude (ca. 115 × 115 m). The bathymetry for the eastern branch of the Whittard Canyon is derived from the GEBCO_2023 Grid, GEBCO Compilation Group (2023) GEBCO 2023 Grid (doi:10.5285/f98b053b-0cbc-6c23-e053-6c86abc0af7b).

## Supporting Information Available

Figures illustrating the details of direct sampling, canyon profiles, microplastic photos, FTIR spectra, grain-size distribution, ^210^Pb dating, and global submarine canyons mapping (Figures S1–S7); tables showing the raw data of microplastic abundance comparison, seafloor sediment samples, contamination control procedural blank, microplastic identification, and Shield’s diagram (Tables S1–S5) (PDF)
