# Supplementary material for: Direct Evidence That Microplastics Are Transported to the Deep Sea by Turbidity Currents
Source: Environ Sci Technol. 2025 Apr 4;59(14):7278–87. doi: 10.1021/acs.est.4c12007 (PMC12004917; doi:10.1021/acs.est.4c12007)
Supplement: Supplementary file 1 — es4c12007_si_001.pdf [file es4c12007_si_001.pdf]

Supporting Information for

Direct evidence that microplastics are transported to the deep sea by turbidity currents

Peng Chen[1,2], Ian A. Kane[1]*, Michael A. Clare[3], Euan L. Soutter[1], Furu Mienis[4], Roy A. Wogelius[1], Edward Keavney[5]

[1]Department of Earth and Environmental Sciences, University of Manchester, Manchester M13 9PL, United Kingdom
[2]School of Earth Sciences and Resources, China University of Geosciences, Beijing 100083, China
[3]Ocean BioGeoscience, National Oceanography Centre, Southampton SO14 3ZH, United Kingdom
[4]Department of Ocean Systems, Royal Netherlands Institute for Sea Research (NIOZ), Den Burg 1790 AB, Netherlands
[5]School of Earth and Environment, University of Leeds, Leeds LS2 9JT, United Kingdom

**Corresponding author:* ian.kane@manchester.ac.uk

Numbers of Figures: 7

Numbers of Tables: 5

Numbers of Pages: 22

Contents

Figures

Figure S1. Pictures showing direct sampling of seafloor sediments and materials suspended by turbidity currents in the Whittard Canyon.

Figure S2. Cross sectional and longitudinal profiles of the canyon.

Figure S3. Representative photos taken by stereomicroscope showing different microfibers and microplastic fragments from the sediment trap.

Figure S4. Representative photos of microfibers and microplastic fragments taken by micro-FTIR (scale: 1 mm side length of a square) and their corresponding polymer types interpreted by FTIR spectra.

Figure S5. Grain size distribution of sediment samples from the seven mono-cores (C1–7) and the sediment trap.

Figure S6. ^{210}Pb activity as a function of depth for box-core 65.

Figure S7. Distribution of the 5849 submarine canyons worldwide as mapped by previous study (modified from *2*).

Tables

Table S1. Comparison of microplastic abundance in different submarine canyons worldwide (see Figure 1E).

Table S2. Details of the seafloor sediment samples, including their core numbers, coordinates, water and sediment depths, original sample and dry weights.

Table S3. Contamination control procedural blank data of the sediment trap (ST) and seafloor sediment samples (C1–7) during sample preparation and microplastic identification.

Table S4. Details of microfibers and microplastic fragments identified in the sediment trap (ST) and seafloor sediment samples (C1–7).

Table S5. Data used for plotting Shield's diagram.

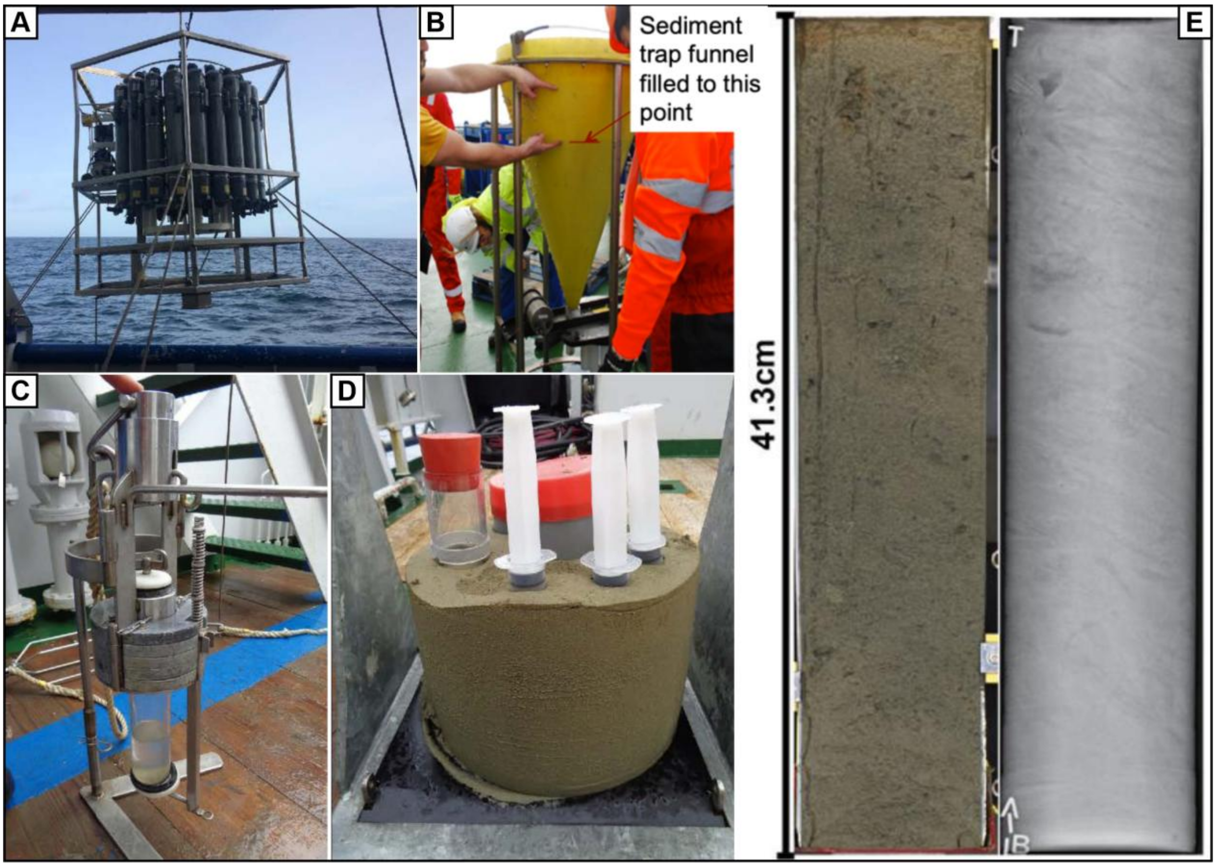

Figure S1. Pictures showing direct sampling of seafloor sediments and materials suspended by turbidity currents in the Whittard Canyon. (A) The Conductivity–Temperature–Depth rosette (CTD) system used to get mono-core samples (see Figure 1). **(B)** The sediment trap used to collect materials suspended by turbidity currents at Mooring M1 (see Figure 1). **(C)** The mono-corer (diameter 6 cm), a small single core device suspended 7 m underneath the CTD. **(D)** A box-core sample with sub-cores (diameter 10 cm) for ^{210}Pb dating, X-ray and grain-size analysis. **(E)** Line-scan image (left) and X-radiograph (right) of box-core 65 (see Figure S6). The silty sediment is homogeneously sorted with signs of bioturbation, and is normally graded from base (B) to top (T).

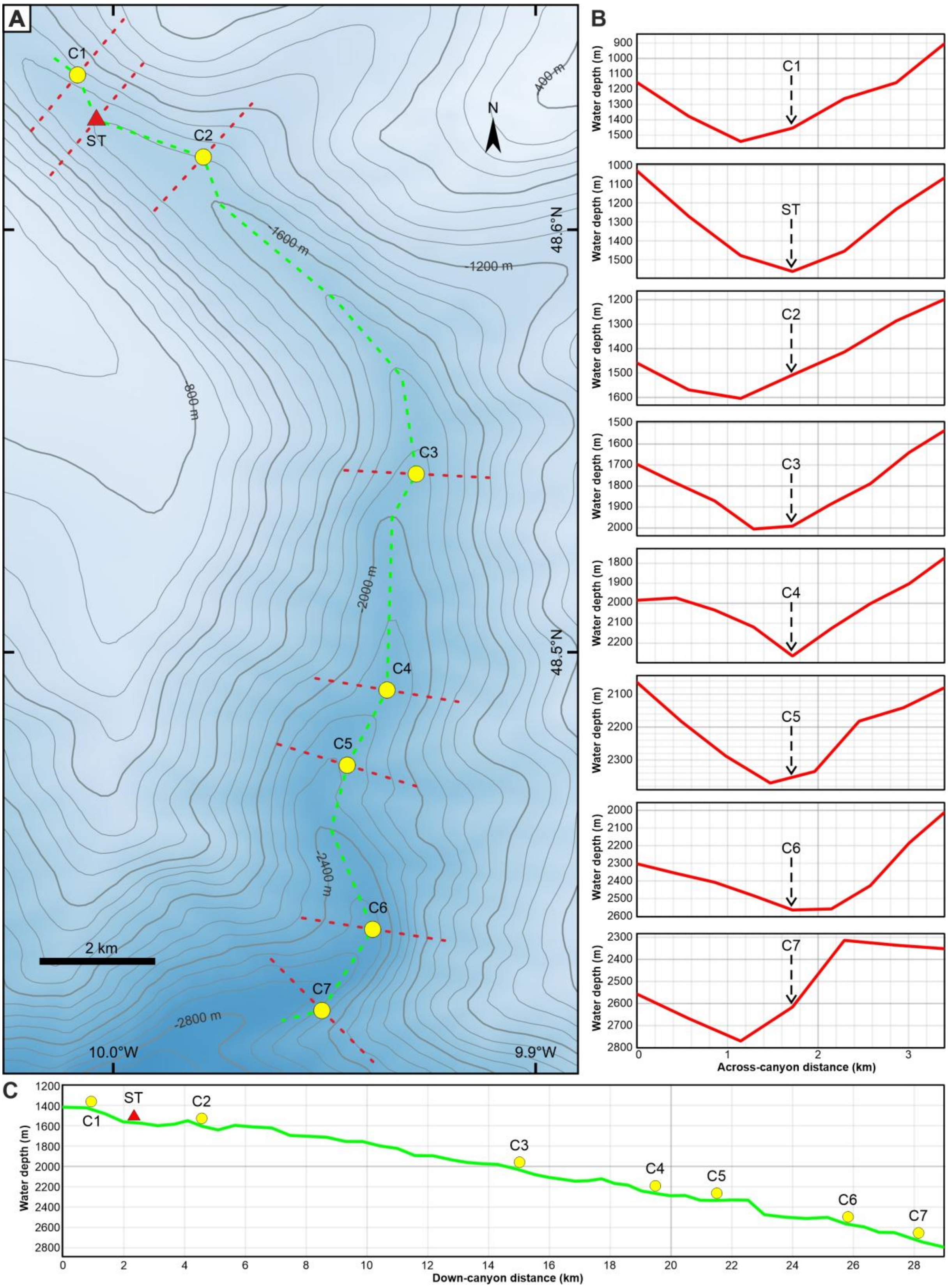

Figure S2. Cross sectional and longitudinal profiles of the canyon. (A) Position of the cross sections and the longitudinal profile. **(B)** Cross sectional geometry of the canyon at each mono-core location (C1–7) and the sediment trap (ST). Note that the mono-core locations are not always at the deepest part of the canyon. **(C)** Longitudinal profile showing the ST and mono-core locations along the canyon.

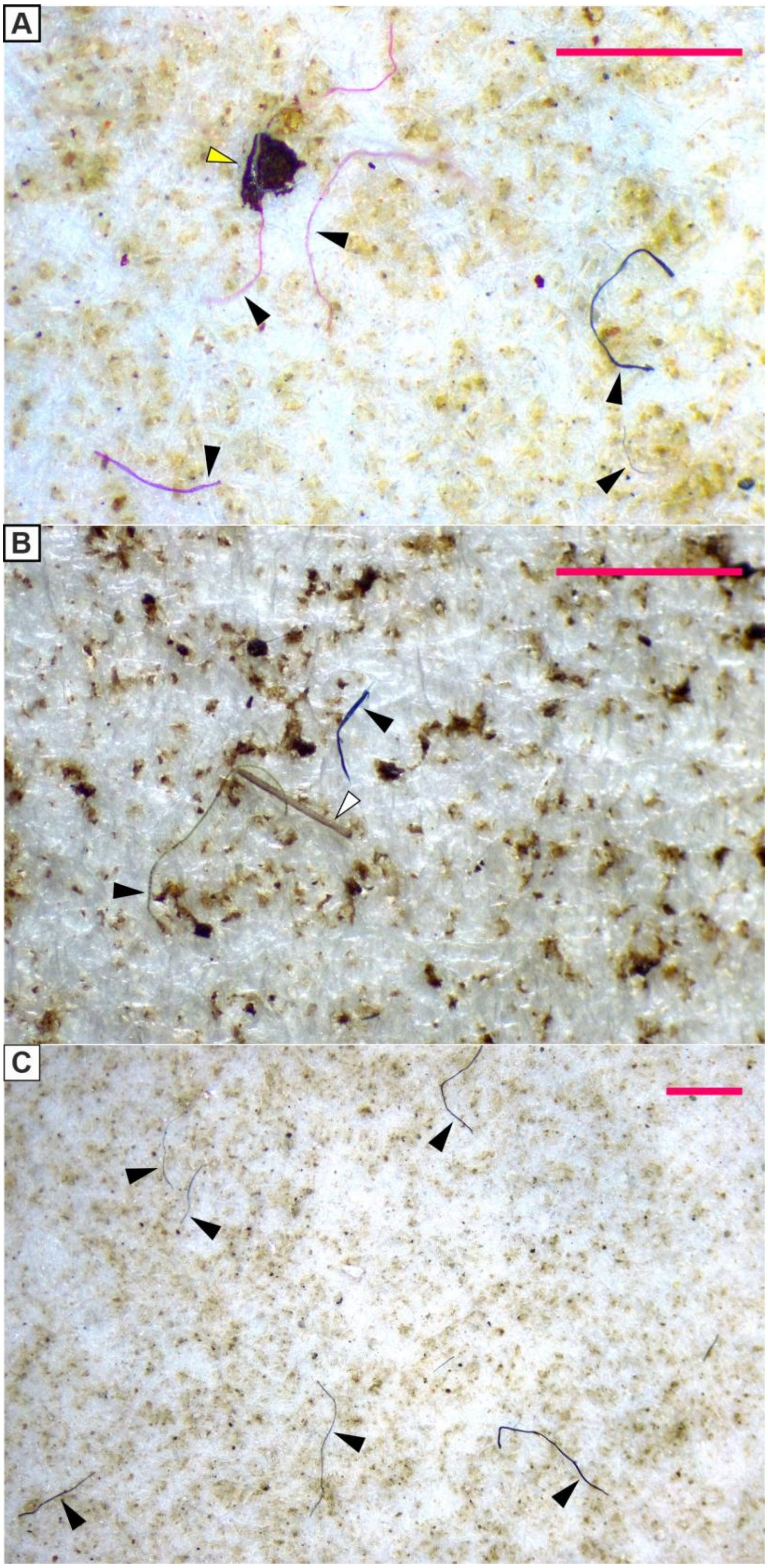

Figure S3. Representative photos taken by stereomicroscope showing different microfibers and microplastic fragments from the sediment trap. Note the background sediments are mainly composed of quartz grains. Black marks, microfibers; yellow mark, microplastic fragment; white mark, phytobenthos. Scale bar 1 mm. **(A)** Pink and blue microfibers, and pink microplastic fragments. **(B)** Blue and gray microfibers, with phytobenthos and organics also shown. **(C)** Blue, gray and purple microfibers.

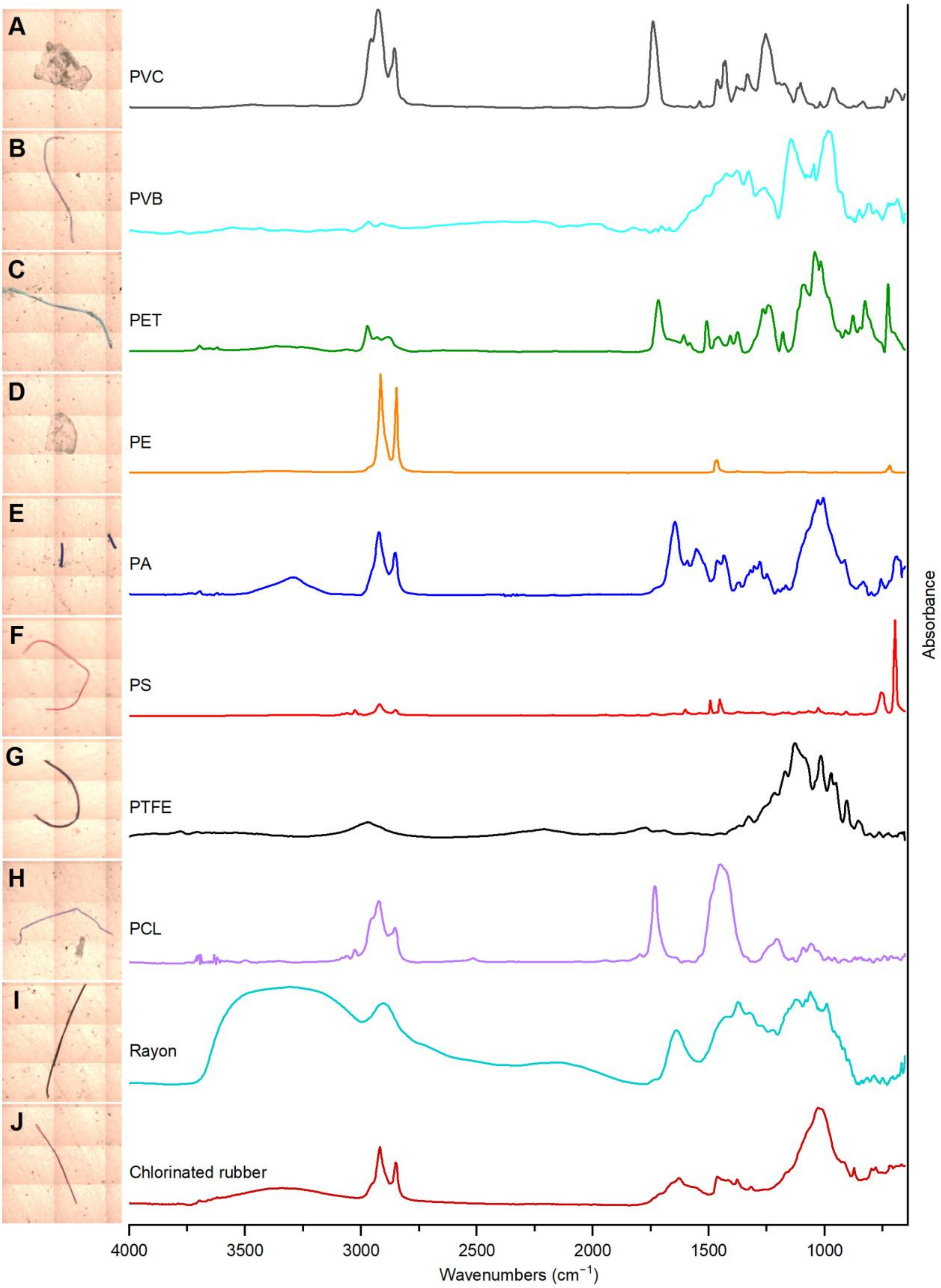

Figure S4. Representative photos of microfibers and microplastic fragments taken by micro-FTIR (scale: 1 mm side length of a square) and their corresponding polymer types interpreted by FTIR spectra. **(A)** PVC (polyvinyl chloride). **(B)** PVB (polyvinyl butyral). **(C)** PET (polyethylene terephthalate). **(D)** PE (polyethylene). **(E)** PA (polyamide). **(F)** PS (polystyrene). **(G)** PTFE (polytetrafluoroethylene). **(H)** PCL (polycaprolactone). **(I)** Rayon. **(J)** Chlorinated rubber.

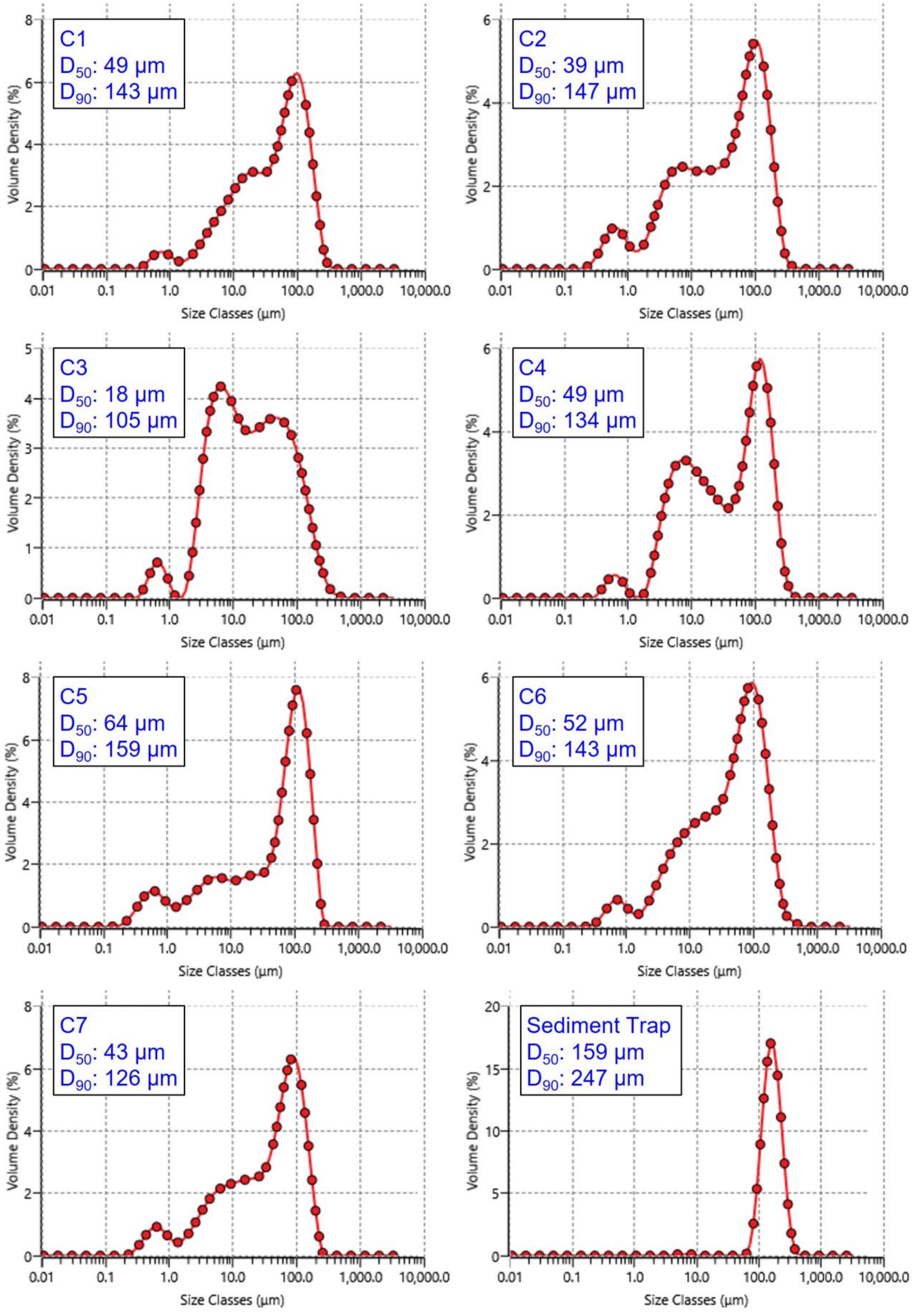

Figure S5. Grain size distribution of sediment samples from the seven mono-cores (C1–7) and the sediment trap. Their mean D_{50} and D_{90} values are shown.

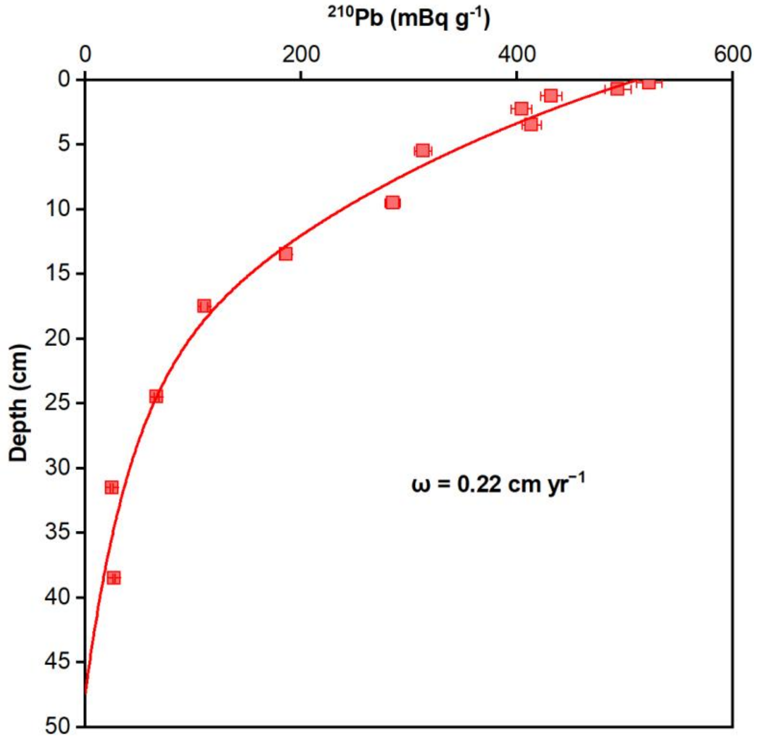

Figure S6. ^{210}Pb activity as a function of depth for box-core 65. Red squares represent the measured ^{210}Pb activities per sediment depth interval, and the gently sloped red profile line represents the modelled ^{210}Pb activity. Sediment accumulation rate is expressed as ω with a value of 0.22 cm yr^{-1} (*1*), indicating the 10 cm sample depth is likely to be deposited entirely within the time range since the first production of plastic in the 1950s.

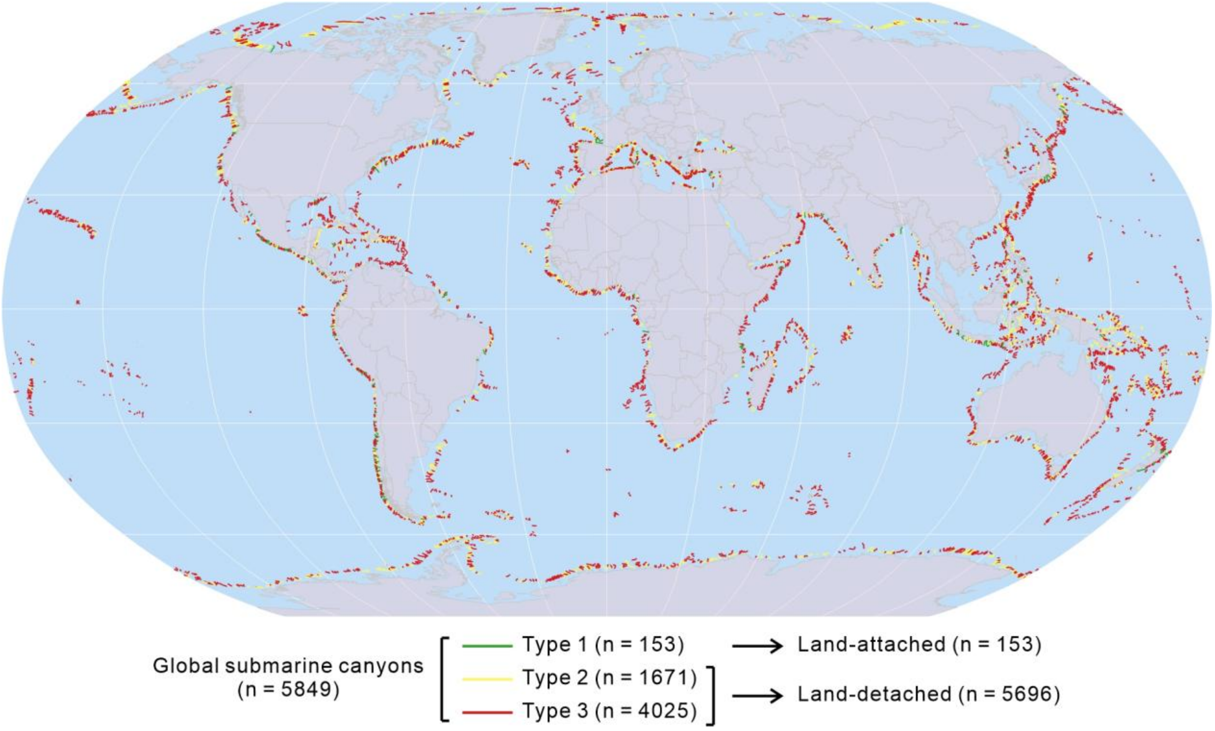

Figure S7. Distribution of the 5849 submarine canyons worldwide as mapped by previous study (modified from *2*). Land-detached submarine canyons are defined as those are not connected to a major river system (Type 2 and Type 3) and account for 97% (n = 5696) of all the submarine canyons worldwide (n = 5849). Note Antarctic canyons are excluded due to a lack of sufficient study.

Table S1. Comparison of microplastic abundance in different submarine canyons worldwide (see Figure 1E). This includes the type, location and sampling depth of each canyon. The mean, standard error of the mean, and maximum microplastic count are all converted to the same unit (items 50 g^{-1} of dried sediment).

Canyon type	Location	Sampling depth (m)	Mean	Standard error of the mean	Maximum
Land-detached	Norfolk (*3*)	196, 197, 572, 573, 810, 819, 1133, 1135	1	0	3
Land-attached	Penghu (*4*)	2727	10		10
Land-detached	Black Sea (*5*)	840	20		20
Land-detached	Gaoping (*4*)	1793, 2712, 2727	8	6	20
Land-detached	Formosa (*4*)	2250	20		20
Land-attached and -detached	Mediterranean (*6*)	300, 1300	23	13	35
Land-detached	NE Atlantic (*6*)	1400, 2000	23	17	40
Land-detached	Alboran Sea (*5*)	389, 407, 609, 932, 1475, 1857	33	5	50
Land-attached and -detached	Cantabrian Sea (*5*)	1207, 2000	55	15	70
Land-attached and -detached	Catalan Sea (*5*)	281, 1190, 1667, 1980, 2064, 2196	48	7	75
Land-detached	Whittard (This Study)	1421, 1581, 1569, 1944, 2270, 2338, 2590, 2683	45	2	78, 82

Table S2. Details of the seafloor sediment samples, including their core numbers, coordinates, water and sediment depths, original sample and dry weights.

Number	Core	Latitude (°)	Longitude (°)	Water depth (m)	Sediment depth (cm)	Original sample weight (g)	Dry weight (g)
1	C1	48.63664	−10.0085	1421	1	21.452	14.2
2	C1	48.63664	−10.0085	1421	2	21.368	14
3	C1	48.63664	−10.0085	1421	3	28.381	24.2
4	C1	48.63664	−10.0085	1421	4	36.381	30
5	C1	48.63664	−10.0085	1421	5	27.041	22.4
6	C1	48.63664	−10.0085	1421	6	32.744	26.8
7	C1	48.63664	−10.0085	1421	7	45.221	34.7
8	C1	48.63664	−10.0085	1421	8	34.436	24.5
9	C1	48.63664	−10.0085	1421	9	29.409	19.2
10	C1	48.63664	−10.0085	1421	10	31.257	20.9
11	C2	48.61722	−9.97869	1569	1	16.196	9.6
12	C2	48.61722	−9.97869	1569	2	23.644	10.7
13	C2	48.61722	−9.97869	1569	3	22.591	12.2
14	C2	48.61722	−9.97869	1569	4	25.958	16.2
15	C2	48.61722	−9.97869	1569	5	26.954	14.6
16	C2	48.61722	−9.97869	1569	6	22.639	14
17	C2	48.61722	−9.97869	1569	7	25.98	16
18	C2	48.61722	−9.97869	1569	8	21.771	12.3
19	C2	48.61722	−9.97869	1569	9	27.508	17.5
20	C2	48.61722	−9.97869	1569	10	20.791	13.5
21	C3	48.54217	−9.92827	1944	1	22.285	22.6
22	C3	48.54217	−9.92827	1944	2	19.191	16.6
23	C3	48.54217	−9.92827	1944	3	15.383	12.5
24	C4	48.49102	−9.9352	2270	1	15.23	13
25	C4	48.49102	−9.9352	2270	2	23.925	18.998
26	C4	48.49102	−9.9352	2270	3	29.711	24.4
27	C4	48.49102	−9.9352	2270	4	23.897	19.8
28	C4	48.49102	−9.9352	2270	5	23.7	17.7
29	C4	48.49102	−9.9352	2270	6	25.069	18.951
30	C4	48.49102	−9.9352	2270	7	23.638	15
31	C4	48.49102	−9.9352	2270	8	35.874	28.9
32	C4	48.49102	−9.9352	2270	9	38.629	32.6
33	C4	48.49102	−9.9352	2270	10	29.696	26.2
34	C5	48.47323	−9.94462	2338	1	37.888	28.6
35	C5	48.47323	−9.94462	2338	2	29.795	20

36	C5	48.47323	−9.94462	2338	3	33.316	27.3
37	C5	48.47323	−9.94462	2338	4	39.354	31.5
38	C5	48.47323	−9.94462	2338	5	48.513	40.5
39	C5	48.47323	−9.94462	2338	6	33.273	23
40	C5	48.47323	−9.94462	2338	7	32.534	22
41	C5	48.47323	−9.94462	2338	8	37.167	29
42	C5	48.47323	−9.94462	2338	9	46.885	40.4
43	C5	48.47323	−9.94462	2338	10	30.776	27
44	C6	48.43438	−9.9386	2590	1	13.094	7
45	C6	48.43438	−9.9386	2590	2	12.692	5.8
46	C6	48.43438	−9.9386	2590	3	14.696	8
47	C6	48.43438	−9.9386	2590	4	20.189	9.5
48	C6	48.43438	−9.9386	2590	5	21.035	10
49	C6	48.43438	−9.9386	2590	6	16.765	11.7
50	C6	48.43438	−9.9386	2590	7	32.125	19.2
51	C6	48.43438	−9.9386	2590	8	32.161	19.6
52	C6	48.43438	−9.9386	2590	9	29.764	19.3
53	C6	48.43438	−9.9386	2590	10	36.744	24.3
54	C7	48.41516	−9.95061	2683	1	27.001	15.5
55	C7	48.41516	−9.95061	2683	2	34.449	24.1
56	C7	48.41516	−9.95061	2683	3	31.075	27.2
57	C7	48.41516	−9.95061	2683	4	35.305	29.9
58	C7	48.41516	−9.95061	2683	5	31.764	26.3
59	C7	48.41516	−9.95061	2683	6	34.188	27.2
60	C7	48.41516	−9.95061	2683	7	37.706	34
61	C7	48.41516	−9.95061	2683	8	37.105	32.3
62	C7	48.41516	−9.95061	2683	9	34.347	28.6
63	C7	48.41516	−9.95061	2683	10	29.081	21.7

Table S3. Contamination control procedural blank data of the sediment trap (ST) and seafloor sediment samples (C1–7) during sample preparation and microplastic identification. The number following the hyphen representing the sediment depth (cm), and N/A corresponding to no data.

Sample number	Sample preparation exposure time (s)	Microfiber count	Microplastic identification exposure time (s)	Microfiber count
ST	429	3	1150	0
C1-1	540	0	920	0
C1-2	515	0	879	0
C1-3	457	0	681	0
C1-4	500	0	534	0
C1-5	492	1	689	0
C1-6	525	0	748	0
C1-7	483	0	503	0
C1-8	471	0	597	0
C1-9	531	0	834	0
C1-10	436	0	412	0
C2-1	388	0	456	0
C2-2	540	0	478	0
C2-3	433	0	502	0
C2-4	420	0	330	0
C2-5	408	0	1347	1
C2-6	403	0	810	0
C2-7	414	1	712	0
C2-8	451	0	629	0
C2-9	439	0	1422	0
C2-10	381	0	878	0
C3-1	430	0	720	0
C3-2	490	0	934	0
C3-3	470	0	701	0
C4-1	467	0	645	0
C4-2	483	0	522	1
C4-3	1962	0	326	0
C4-4	580	0	400	0
C4-5	449	0	354	0
C4-6	552	0	570	0
C4-7	442	0	385	1
C4-8	458	0	380	0
C4-9	415	0	321	0
C4-10	N/A	0	N/A	0
C5-1	N/A	2	N/A	0
C5-2	N/A	0	N/A	0
C5-3	N/A	1	N/A	0
C5-4	N/A	0	N/A	0
C5-5	N/A	2	N/A	1
C5-6	N/A	2	N/A	0
C5-7	N/A	3	N/A	0
C5-8	N/A	3	N/A	1
C5-9	N/A	6	N/A	0
C5-10	421	0	410	0
C6-1	410	0	329	0
C6-2	407	0	717	0
C6-3	467	0	682	0

C6-4	403	0	724	0
C6-5	367	0	520	0
C6-6	366	0	489	0
C6-7	406	0	226	0
C6-8	354	0	460	0
C6-9	362	0	357	0
C6-10	410	0	837	0
C7-1	435	0	869	0
C7-2	391	0	708	0
C7-3	399	0	725	0
C7-4	530	0	713	1
C7-5	406	0	442	1
C7-6	448	4	772	0
C7-7	429	0	729	0
C7-8	954	0	1208	0
C7-9	1065	1	1408	1
C7-10	716	0	528	0

Table S4. Details of microfibers and microplastic fragments identified in the sediment trap (ST) and seafloor sediment samples (C1–7).

Sample	Sediment depth (cm)	Type	Total number	Concentration (items 50 g^{-1} of dried sediment)	Percentage (fibers/ fragments)	Mean concentration in core	Mean percentage (fibers/ fragments) in core	Number of samples selected for FTIR analysis	Polymer types interpreted by FTIR spectra
ST		Fibers	87	74	90.6%	82	90.6%<br>9.4%	2	PET, PVC
ST		Fragments	9	8	9.4%			1	PVC
C1	1	Fibers	11	39	100.0%	41	100.0%<br>0.0%	1	PS
C1	1	Fragments	0	0	0.0%				
C1	2	Fibers	13	46	100.0%				
C1	2	Fragments	0	0	0.0%				
C1	3	Fibers	21	43	100.0%			1	PA
C1	3	Fragments	0	0	0.0%				
C1	4	Fibers	29	48	100.0%				
C1	4	Fragments	0	0	0.0%				
C1	5	Fibers	16	36	100.0%			1	PVB
C1	5	Fragments	0	0	0.0%				
C1	6	Fibers	28	52	100.0%			1	PTFE
C1	6	Fragments	0	0	0.0%				
C1	7	Fibers	17	24	100.0%				
C1	7	Fragments	0	0	0.0%				
C1	8	Fibers	24	49	100.0%			1	PVB
C1	8	Fragments	0	0	0.0%				
C1	9	Fibers	14	36	100.0%				
C1	9	Fragments	0	0	0.0%				
C1	10	Fibers	14	33	100.0%			1	Rayon

C1	10	Fragments	0	0	0.0%				
C2	1	Fibers	11	57	100.0%	43	100.0%<br>0.0%		
C2	1	Fragments	0	0	0.0%				
C2	2	Fibers	11	51	100.0%			1	PE
C2	2	Fragments	0	0	0.0%				
C2	3	Fibers	8	33	100.0%				
C2	3	Fragments	0	0	0.0%				
C2	4	Fibers	12	37	100.0%				
C2	4	Fragments	0	0	0.0%				
C2	5	Fibers	17	58	100.0%			1	PVB
C2	5	Fragments	0	0	0.0%				
C2	6	Fibers	9	32	100.0%			1	PVC
C2	6	Fragments	0	0	0.0%				
C2	7	Fibers	11	34	100.0%				
C2	7	Fragments	0	0	0.0%				
C2	8	Fibers	13	53	100.0%			1	PET
C2	8	Fragments	0	0	0.0%				
C2	9	Fibers	14	40	100.0%			1	PA
C2	9	Fragments	0	0	0.0%				
C2	10	Fibers	8	30	100.0%				
C2	10	Fragments	0	0	0.0%				
C3	1	Fibers	32	71	100.0%	68	100.0%<br>0.0%	1	PVC
C3	1	Fragments	0	0	0.0%				
C3	2	Fibers	22	66	100.0%			1	Chlorinated rubber
C3	2	Fragments	0	0	0.0%				
C3	3	Fibers	17	68	100.0%				

C3	3	Fragments	0	0	0.0%				
C4	1	Fibers	19	73	100.0%	44	92.8%<br>7.2%	1	PVB
C4	1	Fragments	0	0	0.0%				
C4	2	Fibers	22	58	91.7%			1	PET
C4	2	Fragments	2	5	8.3%				
C4	3	Fibers	25	51	100.0%			1	ABS
C4	3	Fragments	0	0	0.0%				
C4	4	Fibers	22	56	91.7%				
C4	4	Fragments	2	5	8.3%				
C4	5	Fibers	18	51	90.0%				
C4	5	Fragments	2	6	10.0%				
C4	6	Fibers	16	42	84.2%			1	PS
C4	6	Fragments	3	8	15.8%				
C4	7	Fibers	11	37	84.6%			1	Rayon
C4	7	Fragments	2	7	15.4%				
C4	8	Fibers	13	22	100.0%				
C4	8	Fragments	0	0	0.0%				
C4	9	Fibers	9	14	90.0%			1	PVC
C4	9	Fragments	1	2	10.0%				
C4	10	Fibers	4	8	100.0%				
C4	10	Fragments	0	0	0.0%				
C5	1	Fibers	30	52	100.0%	40	95.9%<br>4.1%	1	Rayon
C5	1	Fragments	0	0	0.0%				
C5	2	Fibers	16	40	94.1%				
C5	2	Fragments	1	3	5.9%				
C5	3	Fibers	21	38	100.0%				

C5	3	Fragments	0	0	0.0%				
C5	4	Fibers	24	38	100.0%			1	Chlorinated rubber
C5	4	Fragments	0	0	0.0%				
C5	5	Fibers	40	49	88.9%			1	PVC
C5	5	Fragments	5	6	11.1%				
C5	6	Fibers	19	41	95.0%				
C5	6	Fragments	1	2	5.0%				
C5	7	Fibers	12	27	100.0%			1	Rayon
C5	7	Fragments	0	0	0.0%				
C5	8	Fibers	19	33	100.0%			1	PS
C5	8	Fragments	0	0	0.0%				
C5	9	Fibers	19	24	100.0%			1	PE
C5	9	Fragments	0	0	0.0%				
C5	10	Fibers	20	37	87.0%				
C5	10	Fragments	3	6	13.0%				
C6	1	Fibers	7	50	100.0%	47	93.6%<br>6.4%		
C6	1	Fragments	0	0	0.0%				
C6	2	Fibers	9	78	100.0%			1	PTFE
C6	2	Fragments	0	0	0.0%				
C6	3	Fibers	8	50	100.0%				
C6	3	Fragments	0	0	0.0%				
C6	4	Fibers	8	42	80.0%				
C6	4	Fragments	2	11	20.0%				
C6	5	Fibers	10	50	100.0%			1	PET
C6	5	Fragments	0	0	0.0%				
C6	6	Fibers	10	43	90.9%				

C6	6	Fragments	1	4	9.1%				
C6	7	Fibers	15	39	100.0%				
C6	7	Fragments	0	0	0.0%				
C6	8	Fibers	13	33	92.9%				
C6	8	Fragments	1	3	7.1%				
C6	9	Fibers	15	39	78.9%			1	Chlorinated rubber
C6	9	Fragments	4	10	21.1%			1	PA
C6	10	Fibers	7	14	87.5%				
C6	10	Fragments	1	2	12.5%				
C7	1	Fibers	16	52	69.6%	50	83.3%<br>16.7%	1	PVB
C7	1	Fragments	7	23	30.4%			1	PVC
C7	2	Fibers	28	58	90.3%			1	PET
C7	2	Fragments	3	6	9.7%				
C7	3	Fibers	23	42	85.2%				
C7	3	Fragments	4	7	14.8%				
C7	4	Fibers	22	37	84.6%			1	PCL
C7	4	Fragments	4	7	15.4%				
C7	5	Fibers	22	42	81.5%			2	PS, Rayon
C7	5	Fragments	5	10	18.5%				
C7	6	Fibers	22	40	75.9%			1	PA
C7	6	Fragments	7	13	24.1%			1	PE
C7	7	Fibers	23	34	92.0%				
C7	7	Fragments	2	3	8.0%				
C7	8	Fibers	25	39	86.2%				
C7	8	Fragments	4	6	13.8%			1	PVB
C7	9	Fibers	18	31	81.8%				

C7	9	Fragments	4	7	18.2%			1	PET
C7	10	Fibers	18	41	94.7%				
C7	10	Fragments	1	2	5.3%				

Table S5. Data used for plotting Shield's diagram. This includes the maximum velocity (U_{max}) and its corresponding height (h_{max}) of the nine recorded turbidity current profiles from the ADCP in Figure 2D. The particle diameter (D), bed shear stress (τ), Shields parameter (τ_*) and boundary Reynolds number (R_*) of different sediments or plastics are also shown (see Figure 4).

**Sediment/plastic type**	U_{max} **(m s^{-1})**	h_{max} **(mm)**	**D (m)**	**τ (N m^{-2})**	τ_*	R_*
Quartz	3	4000	2.47E-04	10.30	2.62	23.52
Quartz	2.4	5000	2.47E-04	6.35	1.62	18.47
Quartz	2.1	3000	2.47E-04	5.30	1.35	16.87
Quartz	1.9	7000	2.47E-04	3.77	0.96	14.23
Quartz	1.6	1500	2.47E-04	3.47	0.88	13.66
Quartz	1.6	9000	2.47E-04	2.57	0.65	11.75
Quartz	1.3	1000	2.47E-04	2.47	0.63	11.52
Quartz	1.1	1000	2.47E-04	1.77	0.45	9.75
Quartz	1.3	9000	2.47E-04	1.70	0.43	9.54
Quartz	3	4000	1.59E-04	10.30	4.07	15.14
Quartz	2.4	5000	1.59E-04	6.35	2.51	11.89
Quartz	2.1	3000	1.59E-04	5.30	2.10	10.86
Quartz	1.9	7000	1.59E-04	3.77	1.49	9.16
Quartz	1.6	1500	1.59E-04	3.47	1.37	8.79
Quartz	1.6	9000	1.59E-04	2.57	1.02	7.56
Quartz	1.3	1000	1.59E-04	2.47	0.98	7.42
Quartz	1.1	1000	1.59E-04	1.77	0.70	6.28
Quartz	1.3	9000	1.59E-04	1.70	0.67	6.14
Polystyrene (PS)	3	4000	1.00E-03	10.30	49.99	95.21
Polystyrene (PS)	3	4000	1.00E-04	10.30	499.91	9.52
Polystyrene (PS)	1.3	9000	1.00E-03	1.70	8.24	38.64
Polystyrene (PS)	1.3	9000	1.00E-04	1.70	82.36	3.86
Polytetrafluoroethylene (PTFE)	3	4000	1.00E-03	10.30	0.90	95.21
Polytetrafluoroethylene (PTFE)	3	4000	1.00E-04	10.30	8.97	9.52
Polytetrafluoroethylene (PTFE)	1.3	9000	1.00E-03	1.70	0.15	38.64
Polytetrafluoroethylene (PTFE)	1.3	9000	1.00E-04	1.70	1.48	3.86

References

1. J. Kranenburg, F. Mienis, J. H. J. L. van der Lubbe, “The Unknown Role of Whittard Canyon: Pathway or Sink for Organic Carbon.” thesis, Royal Netherlands Institute for Sea Research, (2018).

2. P. T. Harris, T. Whiteway, Global distribution of large submarine canyons: Geomorphic differences between active and passive continental margins. *Mar. Geol.* **285**, 69–86 (2011).

3. E. S. Jones, S. W. Ross, C. M. Robertson, C. M. Young, Distributions of microplastics and larger anthropogenic debris in Norfolk Canyon, Baltimore Canyon, and the adjacent continental slope (Western North Atlantic Margin, USA). *Mar. Pollut. Bull*. **174**, 113047 (2022).

4. X. Zhang, Z. Liu, Y. Zhao, P. Ma, C. Colin, A. T. Lin, Distribution and controlling factors of microplastics in surface sediments of typical deep-sea geomorphological units in the northern South China Sea. *Front. Mar. Sci.* **9**, 1047078 (2022).

5. A. Sanchez-Vidal, R. C. Thompson, M. Canals, W. P. de Haan, The imprint of microfibres in southern European deep seas. *PLoS One* **13**, e0207033 (2018).

6. L. C. Woodall, A. Sanchez-Vidal, M. Canals, G. L. Paterson, R. Coppock, V. Sleight, A. Calafat, A. D. Rogers, B. E. Narayanaswamy, R. C. Thompson, The deep sea is a major sink for microplastic debris. *R. Soc. Open Sci.* **1**, 140317 (2014).
